# Supplementary material for: Anticoagulation Monitoring with Activated Partial ThromboPlastin Time and Anti-Xa Activity in Intensive Care Unit Patients: Interest of Thrombin Generation Assay
Source: Int J Mol Sci. 2022 Sep 23;23(19):11219. doi: 10.3390/ijms231911219 (PMC9570449; doi:10.3390/ijms231911219)
Supplement: Supplementary file 1 [file ijms-23-11219-s001.zip › ijms-1913622-Supplementary .pdf]

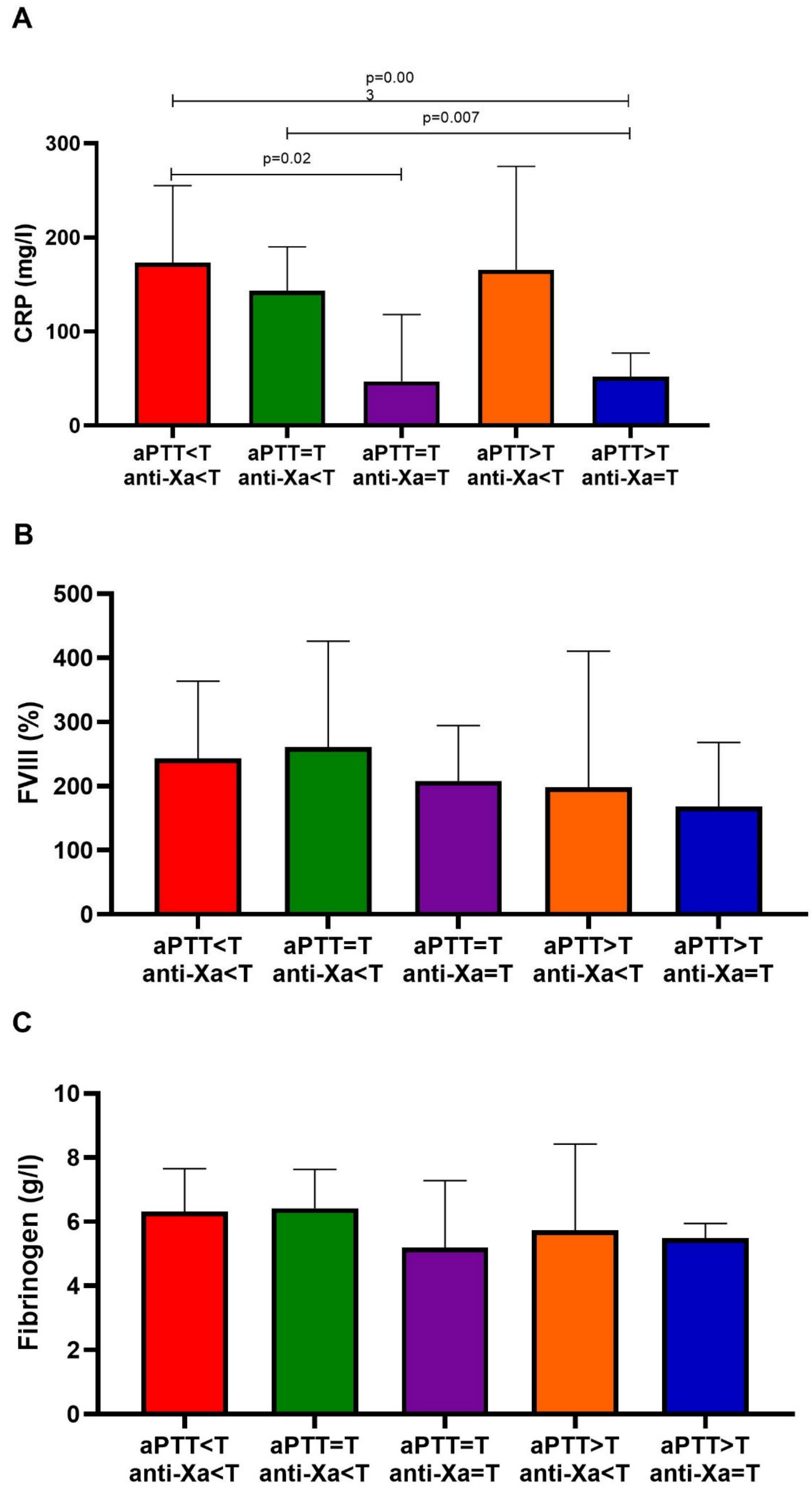

Supplementary Figure S1. Comparison between inflammatory biomarkers and anticoagulation group. Comparison with CRP (**A**), FVIII (**B**), and fibrinogen (**C**). CRP: C-reactive protein. FVIII: factor

VIII. Comparison was performed with Kruskal–Wallis ANOVA with Dunn’s multiple comparisons post-test.

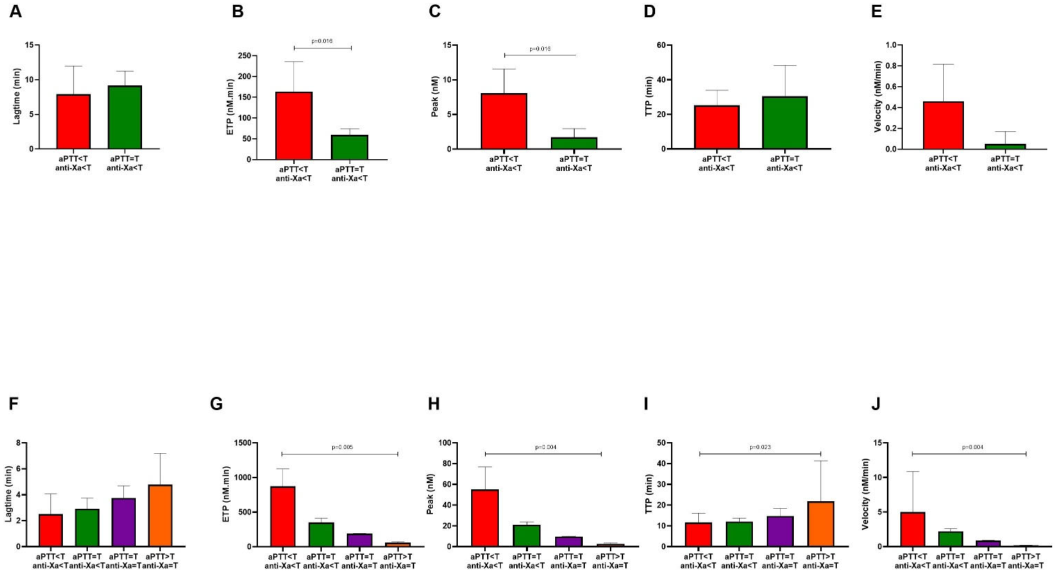

Supplementary Figure S2. Comparison between thrombin generation parameters and anticoagulation group. Comparison of anticoagulation group with 5 pM of tissue factor for lagtime (**A**), ETP (**B**), peak (**C**), time to peak (**D**), and velocity (**E**). Comparison of anticoagulation group with 20 pM of tissue factor for lagtime (**F**), ETP (**G**), peak (**H**), time to peak (**I**) and velocity (**J**). Comparison was performed with Kruskal–Wallis ANOVA with Dunn’s multiple comparisons post-test or Mann–Whitney test.

Table S1. Correlation between thrombin generation parameters, anticoagulant monitoring, and CRP. Results are presented as Pearson correlation coefficient. aPTT: activated partial thromboplastin time. CRP: C-Reactive Protein. ETP: Endogenous Thrombin Potential.

	Lagtime	Time to Peak	ETP	Peak
**aPTT**	**5 pM of tissue factor**			
	0.19 [−0.05; 0.42]	0.46 [0.24; 0.64]	−0.32 [−0.53; −0.08]	−0.45 [−0.62; −0.22]
**Anti-Xa**	**5 pM of tissue factor**			
	0.39 [0.16; 0.58]	0.70 [0.55; 0.81]	−0.61 [−0.75; −0.43]	−0.62 [−0.75; −0.44]
**CRP**	**5 pM of tissue factor**			
	−0.38 [−0.58;−0.13]	−0.42[−0.62; −0.18]	0.57 [0.36; 0.67]	0.51 [0.28; 0.67]
**aPTT**	**20 pM of tissue factor**			
	0.08 [−0.14; 0.29]	0.52 [0.0.34; 0.66]	−0.53 [−67; −0.35]	−0.22 [−0.42; −0.01]
**Anti-Xa**	**20 pM of tissue factor**			
	0.004 [−0.21; 0.22]	0.72 [0.60; 0.81]	−0.64 [−0.74; −0.51]	−0.36 [−0.52; −0.18]
**CRP**	**20 pM of tissue factor**			
	−0.20 [−0.44; 0.06]	−0.40 [−0.60;−0.17]	0.44 [0.20; 0.62]	0.56 [0.36; 0.71]

Table S2. Comparison with 50 % ETP reduction. N: number of patient samples in each group. n: number of patient samples with increased ETP. aPTT: activated partial thromboplastin time. ETP: Endogenous Thrombin Potential. [a] comparison between aPTT < T and Anti-Xa < T and aPTT = T and Anti-Xa < T. [b] comparison between aPTT = T and Anti-Xa < T and aPTT = T and Anti-Xa = T. [c]comparison between aPTT = T and Anti-Xa = T and aPTT > T and Anti-Xa < T. [d] comparison between aPTT > T and Anti-Xa < T and aPTT > T and Anti-Xa = T. Comarison with chi square test.

Group	UFH Target	TGA Condition	ETP Outside Range	n	p
ICU	aPTT < T	5 pM	ETP > 672nM.min	21	
(N = 27)	Anti-Xa < T	20 pM	ETP > 831 nM.min	25	
ICU	aPTT = T	5 pM	ETP > 672nM.min	22	0.0139 [a]
(N = 47)	Anti-Xa < T	20 pM	ETP > 831nM.min	28	0.0028 [a]
CU	aPTT = T	5 pM	ETP > 672nM.min	0	0.0084 [b]
(N = 9)	Anti-Xa = T	20 pM	ETP > 831 nM.min	0	0.018 [b]
ICU	aPTT > T	5 pM	ETP > 672nM.min	0	0.3077 [c]
(N = 4)	Anti-Xa < T	20 pM	ETP > 831nM.min	1	0.99 [c]
ICU	aPTT > T	5 pM	ETP > 672nM.min	0	0.99 [d]
(N = 12)	Anti-Xa = T	20 pM	ETP > 831 nM.min	0	0.99 [d]
